# Prevalence and risk factors of eligibility for anti-osteoporosis medication in a cohort of men ≥50 years and postmenopausal women who had already experienced bariatric surgery

**DOI:** 10.1530/EC-25-0368

**Published:** 2025-10-07

**Authors:** Thomas Flament, Hélène Verkindt, Léa Mortain, François Pattou, Julien Paccou

**Affiliations:** ^1^ CHU Lille, Rheumatology Department, Lille, France; ^2^ CHU Lille, Endocrine and Metabolic Surgery Department, Lille, France; ^3^ CHU Lille, Biostatistics Department, Lille, France; ^4^ University Lille, CHU Lille, MABlab ULR 4490, Rheumatology Department, Lille, France

**Keywords:** bone, calcium, obesity

## Abstract

The 2022 recommendations of the European Calcified Tissue Society (ECTS) suggest initiating anti-osteoporotic medication (AOM) in case of a T-score ≤ −2 and/or in case of a fragility fracture within less than 2 years. Therefore, this study aimed to evaluate the eligibility for AOM in a cohort of patients referred for bone health assessment after bariatric surgery. This observational, cross-sectional, and monocentric study conducted at Lille University Hospital evaluated the prevalence of AOM eligibility according to the ECTS criteria in postmenopausal women and men aged ≥50 years referred for bone health assessment after bariatric surgery, either Roux-en-Y gastric bypass or sleeve gastrectomy, at least 2 years after bariatric surgery. Between June 2019 and June 2023, all participants were referred for bone health assessment, including systematic screening using dual-energy X-ray (DXA) and a standardized questionnaire by a radiology technician. Data between June 2023 and May 2024 were retrospectively reviewed. Among 140 patients (120 women, with an average age of 59 (55–63) years) seen for bone health assessment between June 2019 and June 2023, 33 met the ECTS guidelines for AOM, indicating a prevalence of 24% (CI 95%: 17–31%). Most patients met the BMD T-score ≤ −2 criterion (*n* = 26/140, 19% (CI 95%: 12–25%)) and/or had a recent fragility fracture history (*n* = 14/140, 10% (CI 95%: 5–15%)). In this study, one-fourth of the participants were eligible for AOM according to the ECTS guidelines.

## Introduction

Recent advancements in surgical procedures for people with obesity (PwO) have resulted in significant weight loss and improvements or resolution of certain obesity-associated health conditions (1, 2). However, these procedures are also associated with adverse effects on bone health, including high-turnover bone loss and a higher risk of fractures (3, 4, 5).

The European Calcified Tissue Society (ECTS) recently issued guidelines for preventing and treating osteoporosis secondary to bariatric surgery (6). Anti-osteoporosis medication (AOM) is recommended for men aged ≥50 and postmenopausal women, both before and following surgery, if they meet any of the following criteria: i) Bone mineral density (BMD) T-score ≤ −2 at any measurement site, ii) history of recent fragility fracture after age 40, and iii) FRAX® score ≥3% for hip fractures and/or ≥20% for 10-year major osteoporotic fractures (6).

A retrospective cohort study was carried out to assess the application of the ECTS 2022 recommendations for screening PwO eligible for AOM in the context of bariatric surgery (7). In a cohort of 170 participants, Courtalin *et al.* found that approximately 20% of the population met the ECTS criteria, indicating a prevalence of 19.6% (CI 95%: 13.9–26.5%), with no significant difference between patients expecting bariatric surgery (*n* = 96) and those who had already experienced the procedure (*n* = 74) (7).

However, a major limitation was the pooling of patients expecting surgery with those who had already experienced it, as well as combining all types of procedures. Considering these limitations, a new study was conducted to assess the prevalence of AOM eligibility among patients who had experienced surgery, with a minimum of 2 years of follow-up after the procedure. We focused exclusively on patients who underwent sleeve gastrectomy (SG) or Roux-en-Y gastric bypass (RYGB). In addition, we thoroughly evaluated the risk factors associated with AOM eligibility.

This research aimed to evaluate the prevalence and risk factors of AOM eligibility according to the ECTS recommendations in a cohort of participants who had already experienced bariatric surgery.

## Patients and methods

### Design

**Figure 1:**
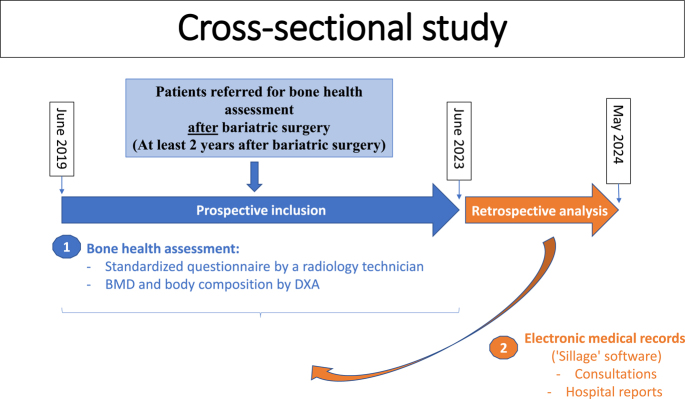
Study design.

Single-center, descriptive, cross-sectional study conducted at Lille University Hospital. As routine practice, the patients were prospectively enrolled between June 2019 and June 2023. Data were analyzed retrospectively between June 2023 and May 2024. The study included patients referred from the Department of Metabolic Surgery to the Rheumatology Department for bone health assessment after bariatric surgery (Fig. 1). As the review of the data was done retrospectively, obtaining written consent was not required. Ethical approval was obtained for this research by the local ethics committee.

### Population

The inclusion criteria encompassed men aged 50 years or older and postmenopausal women, who were under follow-up at Lille University Hospital. These were PwO who were assessed postoperatively following a previous bariatric surgery procedure, either RYGB or SG, with a minimum period of 2 years between surgery and bone health assessment. Exclusion criteria included patients who had not performed DXA at Lille University Hospital, those with one or more pathologies that made it technically challenging to accurately evaluate BMD (e.g., severe hip dysplasia, bilateral hip prosthesis, or lumbar spine surgery), and PwO >160 kg, which is the maximum weight limit for the table.

### Protocol

During the evaluation at the Department of Rheumatology, patients were systematically invited to undergo a DXA scan to assess their hip and lumbar spine BMD. During this assessment, a radiology technician collected various types of data using a standardized questionnaire. This data included information on medications, such as proton pump inhibitors (PPI), history of fragility fractures, and risk factors for osteoporosis. A physician from the Department of Rheumatology then interpreted the DXA results and contacted patients by phone if any data, particularly history of fracture, were incomplete or missing. In addition, comorbidities such as obstructive sleep apnea (OSA), chronic malnutrition, hypertension, and type 2 diabetes were also recorded from i) the questionnaire completed by the radiology technician during the DXA assessment and ii) electronic medical records accessed via the ‘Sillage’ software of Lille University Hospital.

#### Bone mineral density and body composition by DXA

BMD, measured in g/cm^2^, was assessed at the non-dominant hip and the lumbar spine (L1–L4) using a DXA machine (HOLOGIC Discovery A S/N 81360). To evaluate body composition parameters, we adhered to the Adult Official Positions of the International Society for Clinical Densitometry (ISCD), updated in 2023 (8). All patients received total-body DXA scans (HOLOGIC Discovery A S/N 81360). Appendicular lean mass (ALM, kg) was calculated by summing the lean tissue compartments of both arms and legs. The skeletal muscle mass index (SMI) was determined by dividing ALM by height squared (kg/m^2^).

#### History of recent fragility fracture

History of fragility fracture within less than 2 years was systematically collected by questioning during the interview with our radiology technician. If history of fracture was incomplete or missing, a physician from the Department of Rheumatology (JP) contacted patients by phone when interpreting the DXA results. In addition, we opportunistically searched for vertebral fractures on scans performed within the last 5 years, particularly abdominal and pelvic scans if these were available.

#### Biological data

We collected the following biological parameters if they were available from the Metabolic Surgery Department: 25(OH) vitamin D and intact parathyroid hormone (iPTH).

### Eligibility to AOM

Men over the age of 50 and postmenopausal women were recommended to take AOM following bariatric surgery if they met any of the following conditions: i) they had experienced a recent fragility fracture (within the past 2 years) after turning 40, and ii) their BMD T-score was ≤ −2 at any measurement site. Since no patients in the previous study (7) met the FRAX® criterion alone, this criterion was not included in this study.

### Statistical analysis

Categorical variables are expressed as counts (percentages), whereas quantitative variables are represented as either the mean (standard deviation, SD) or the median (interquartile range, IQR) for distributions that are not Gaussian. The relationships between eligibility for AOM and prespecified risk factors were assessed using univariate logistic regression analysis comparing eligible versus non-eligible patients. Log-linearity was assessed with restricted cubic splines for quantitative variables; the ‘time between bariatric surgery and DXA’ was dichotomized at the median due to violation of this assumption. Statistical tests were conducted with a two-tailed α level of 0.05. The data analysis was performed using SAS software (version 9.4; SAS Institute, USA).

## Results

In this group of 140 patients, comprising 120 women (86%), the average age was 59 years, with an SD of 6.1 years. The average body mass index (BMI) was 32.8 kg/m^2^, with an SD of 7.0. The baseline patient characteristics are shown in Table 1. All participants underwent DXA during the postoperative follow-up, with 79% having experienced RYGB and 21% SG. The median time from surgery to DXA was 5 years (IQR: 2–8 years). The mean 25(OH) vitamin D level across the cohort was low, averaging 27.0 ng/mL, with an SD of 9.3. At the time of the DXA scan, fewer than half of the patients reported taking calcium or vitamin D supplements, and there was no evidence of prior or current AOM use. In terms of recent fragility fractures, 19 fractures were recorded in 14 patients, with the proximal humerus being the most common site (*n* = 5), followed by the wrist/forearm (*n* = 4), and the ankle/leg (*n* = 3) (Table 2). Of the 140 patients, 33 met the ECTS criteria for AOM eligibility, representing a prevalence of 24% (CI 95%: 17–31%). Most patients qualified based on a BMD T-score ≤ −2 (*n* = 26, 19% (CI 95%: 12–25%)) and/or a recent fragility fracture history (*n* = 14, 10% (CI 95%: 5–15%)).

**Table 1:** Patients’ general characteristics at the time of dual-energy X-ray absorptiometry (DXA).

	Total
	(*n* = 140)
Age (years)	59.0 ± 6.1
Women	120/140 (85.7)
Weight (kg)	89.3 ± 21.5
Height (cm)	164.6 ± 8.4
Body mass index (kg/m^2^)	32.8 ± 7.0
Time from surgery (years)	5.0 (2.0; 8.0)
Comorbidities	
Hypertension	83/138 (60.1)
Obstructive sleep apnea	83/139 (59.7)
Chronic malnutrition	53/140 (37.9)
Type 2 diabetes	49/140 (35.0)
Osteoporosis risk factors	
Excessive alcohol consumption (weaned or not)	19/140 (13.6)
Smoking (weaned or not)	62/139 (44.6)
Early menopause	26/103 (25.2)
Family history of hip fracture	5/138 (3.6)
Bone mineral density by DXA	
Lumbar spine (T-score)	0.0 ± 1.6 (*n* = 139)
Total hip (T-score)	−0.3 ± 1.1 (*n* = 136)
Femoral neck (T-score)	−0.8 ± 1.1 (*n* = 136)
Body composition by DXA	
Total body fat (%)	46.5 ± 7.6 (*n* = 134)
Appendicular lean mass (kg)	20.7 ± 5.3 (*n* = 130)
Skeletal muscle index (kg/m^2^)	6.3 (5.5; 7.1) (*n* = 134)
Biological tests	
25(OH) vitamin D (ng/mL)	27.0 ± 9.3 (*n* = 120)
Serum intact PTH (pg/mL)	43.0 (35.0; 68.0) (*n* = 119)

Values expressed as number (%), mean ± SD, or median (IQR).

Abbreviations: SD, standard deviation; IQR, interquartile range; PTH, parathyroid hormone.

**Table 2:** History of recent fragility fracture (within less than 2 years).

N	Age at DXA evaluation (years)	Type of surgery	Type of fragility fractures	Time from surgery (years)
		(date)	(date)	
1	Men, 56	SG	Lower leg	2
	(2020)	(2017)	(2019)	
2	Women, 57	RYGB	Proximal humerus (2019)	14
	(2021)	(2005)	Wrist/forearm (2019)	
3	Women, 48	RYGB	Wrist/forearm	8
	(2019)	(2009)	(2017)	
4	Women, 57	RYGB	Ankle	4
	(2021)	(2016)	(2020)	
5	Women, 58	RYGB	Vertebrae (T11, L1, and L3)	9
	(2020)	(2011)	(2020)	
6	Women, 69	RYGB	Proximal humerus (2019)	6
	(2019)	(2013)	Hip (2019)	
			Vertebrae (L4) (2019)	
7	Women, 71	SG	Pelvis	5
	(2019)	(2012)	(2017)	
8	Women, 70	RYGB	Ankle	10
	(2020)	(2010)	(2020)	
9	Women, 52	RYGB	Proximal humerus	7
	(2022)	(2014)	(2021)	
10	Women, 49	RYGB	Wrist/forearm	6
	(2022)	(2016)	(2022)	
11	Women, 52	SG	Proximal humerus	3
	(2022)	(2019)	(2022)	
12	Women, 63	RYGB	Rib	10
	(2023)	(2013)	(2023)	
13	Women, 60	RYGB	Wrist/forearm	10
	(2023)	(2011)	(2021)	
14	Men, 67	RYGB	Proximal humerus	4
	(2023)	(2017)	(2021)	

SG, sleeve gastrectomy; RYGB, Roux-en-Y gastric bypass.

Table 3 presents the univariate analysis results for the prespecified risk factors and AOM eligibility. The two risk factors linked to AOM eligibility were age (odds ratio (OR) = 1.08 (1.01; 1.16)) and iPTH (OR = 1.02 (1.001; 1.03)). Each additional year of age was associated with an 8% higher risk of AOM eligibility, while each 1 pg/mL increase in iPTH corresponded to a 2% increase in eligibility risk for AOM.

**Table 3:** Relationships between prespecified risk factors and treatment eligibility according to ECTS recommendations.

	Not eligible for AOM	Eligible for AOM	Odds ratio	*P*-value
	(*n* = 107)	(*n* = 33)		
Gender				
Women	92/107 (86.0)	28/33 (84.8)		
Men	15/107 (14.0)	5/33 (15.2)	1.10 (0.37; 3.28)	0.87
Smoking (weaned or not)				
No	47/106 (44.3)	15/33 (45.5)		
Yes	59/106 (55.7)	18/33 (54.5)	1.05 (0.48; 2.29)	0.91
Type of bariatric surgery				
Gastric bypass	84/107 (78.5)	26/33 (78.8)		
Sleeve gastrectomy	23/107 (21.5)	7/33 (21.2)	0.98 (0.38; 2.55)	0.97
Excessive alcohol intake (weaned or not)				
No	95/107 (88.8)	26/33 (78.8)		
Yes	12/107 (11.2)	7/33 (21.2)	2.13 (0.76; 5.96)	0.15
Time between BMD evaluation by DXA and bariatric surgery**^†^**				
<5 years	47/107 (43.9)	9/33 (27.3)		
≥5 years	60/107 (56.1)	24/33 (72.7)	2.09 (0.89; 4.92)	0.09
PPI treatment				
No	73/106 (68.9)	21/33 (63.6)		
Yes	33/106 (31.1)	12/33 (36.4)	1.26 (0.56; 2.87)	0.58
Type 2 diabetes				
No	72/107 (67.3)	19/33 (57.6)		
Yes	35/107 (32.7)	14/33 (42.4)	1.52 (0.68; 3.37)	0.31
Chronic malnutrition				
No	66/107 (61.7)	21/33 (63.6)		
Yes	41/107 (38.3)	12/33 (36.4)	0.92 (0.41; 2.07)	0.84
Age	58.0 (54.0; 63.0)	61.0 (57.0; 65.0)	1.08 (1.01; 1.16)^‡^	0.028
Body mass index (kg/m^2^)	32.4 (28.6; 38.1)	32.0 (25.6; 35.6)	0.96 (0.90; 1.02)^‡^	0.16
Skeletal muscle index (kg/m^2^)	6.3 (5.7; 7.1) (*n* = 105)	5.5 (5.0; 7.1) (*n* = 29)	0.82 (0.59; 1.15)^‡^	0.25
Serum intact PTH (pg/mL)	42.0 (35.0; 60.0) (*n* = 91)	67.5 (35.0; 89.5) (*n* = 28)	1.02 (1.001; 1.03)^‡^	0.033

IQR, interquartile range; SD, standard deviation. Values expressed as number (%) or median (IQR).

OR calculated for eligible for AOM vs not eligible for AOM.

^†^ Time between BMD evaluation by DXA and bariatric surgery was dichotomized according to the median, as the assumption of log-linearity was not met.

^‡^ OR per one-unit increase.

## Discussion

This study represents the second investigation into the use of the ECTS 2022 guidelines for preventing and treating osteoporosis secondary to bariatric surgery. This study assessed the prevalence and risk factors for AOM eligibility in a group of patients who had experienced bariatric surgery (RYGB or SG) with at least 2 years of follow-up. Our findings revealed that a significant proportion of the patients (*n* = 33, 24%) qualified for AOM. In addition, age and iPTH levels were identified as the risk factors associated with AOM eligibility.

Our findings are consistent with those of a previous investigation (7). However, there is an overlap between the two populations, which introduces selection bias. The results demonstrated that the BMD T-score ≤ −2 criterion played a crucial role in treatment decisions, as many patients (*n* = 26) were eligible for therapy based on their BMD scores. This highlights the necessity of regularly performing this test in the target population, particularly in those who have experienced bariatric surgery, as it is both practical and advantageous for such procedures. According to ECTS guidelines, DXA should be performed routinely and preoperatively in postmenopausal women and men aged ≥50 years. In patients who do not require AOM, a follow-up with DXA should be performed at 2–3 years after surgery, or sooner should a fragility fracture occur in the interval (6).

Furthermore, a history of recent fragility fractures after the age of 40 years was another key criterion influencing treatment decisions for 14 patients. The most common fracture site was the proximal humerus, followed by the wrist/forearm and ankle/leg, which is consistent with the literature. Fracture sites such as the ankle/leg and proximal humerus are commonly associated with obesity, and wrist fractures represent one of the most common types of fragility fractures following bariatric surgery, especially for RYGB (9, 10, 11).

Age and iPTH level were two risk factors associated with AOM eligibility. This discovery highlights the need to assess the long-term effects of different bariatric surgeries on bone health in postmenopausal women and men aged ≥50 years, especially in patients aged ≥60 years, which is an age limit particularly at risk of complications after bariatric surgery (12, 13). In addition to age, elevated iPTH levels have emerged as a risk factor for AOM eligibility. This finding aligns with the central role of iPTH in bone metabolism: chronic elevation, particularly in secondary hyperparathyroidism, promotes bone resorption and compromises both bone density and quality (14). This likely reflects both the relatively low levels of vitamin D in the population and insufficient calcium and vitamin D supplementation. According to the French guidelines (4), in all patients with an indication for or who have already undergone bariatric surgery, iPTH should be measured and assessed on a regular basis so that prescriptions of calcium and vitamin D supplementation can be adjusted. Moreover, in the first year after bariatric surgery, a biological work-up to assess serum calcium, phosphate, 25(OH) vitamin D, and iPTH is recommended every 3 months to maintain an optimal level of vitamin D and calcium supplementation and rule out secondary hyperparathyroidism (4). More extensive studies with larger numbers of participants are required to thoroughly evaluate the risk factors related to AOM eligibility.

Our study has several notable strengths, such as i) consistency in BMD measurements, which were conducted using the same machine by a single radiology technician and interpreted by a rheumatologist, both of whom had extensive experience with PwO, and ii) a thorough review of all medical records by a single investigator. Instances of missing data were rare. Our study has several limitations, including its cross-sectional nature. Nonetheless, patients were included prospectively, and data analyses were conducted retrospectively. We also acknowledge that relying on self-reported fracture history could have resulted in misclassifications. The lack of a control group prevented us from drawing definitive conclusions regarding the high percentage of patients eligible for AOM post-bariatric surgery. In addition, we chose not to include the FRAX calculation to assess the eligibility for AOM after bariatric surgery, which may be considered a limitation. Finally, the single-center design limited the generalizability of our findings. However, the participants in this study represent a well-defined group for osteoporosis, typically monitored in specialized obesity centers, such as Lille University Hospital. Ultimately, we cannot rule out the possibility that our group of patients represents a high-risk cohort, as not all individuals were referred by the Department of Metabolic Surgery for bone health assessment, which may have introduced selection bias.

In summary, we found that one-fourth of our selected population, referred for bone health evaluation after bariatric surgery, met the ECTS criteria for AOM. Further research is necessary to assess the effectiveness and safety of AOM in managing high-turnover bone loss and osteoporosis in this population (15, 16, 17). In accordance with the ECTS guidelines (6), in patients who require AOM, the parenteral route should be favored, as it affords the most appropriate absorption. Zoledronic acid should be used as the first line of treatment (15, 16, 17).

## Declaration of interest

Thomas Flament, Hélène Verkindt, Léa Mortain, François Pattou, and Julien Paccou declare that they have no conflicts of interest.

## Funding

This study received no external funding. The authors are employed by their university and/or their hospital. These funding organizations did not suggest the subject of this study and did not have access to the results before publication.

## References

[1] Arterburn DE, Telem DA, Kushner RF, et al. Benefits and risks of bariatric surgery in adults: a review. JAMA 2020 324 879–887. (10.1001/jama.2020.12567) 32870301

[2] Courcoulas AP, Patti ME, Hu B, et al. Long-term outcomes of medical management vs bariatric surgery in type 2 diabetes. JAMA 2024 331 654–664. (10.1001/jama.2024.0318) 38411644 PMC10900968

[3] Paccou J, Caiazzo R, Lespessailles E, et al. Bariatric surgery and osteoporosis. Calcif Tissue Int 2022 110 576–591. (10.1007/s00223-020-00798-w) 33403429

[4] Paccou J, Genser L, Lespessailles É, et al. French recommendations on the prevention and treatment of osteoporosis secondary to bariatric surgery. Joint Bone Spine 2022 89 105443. (10.1016/j.jbspin.2022.105443) 35908644

[5] Gagnon C & Schafer AL. Bone health after bariatric surgery. JBMR Plus 2018 2 121–133. (10.1002/jbm4.10048) 30283897 PMC6124196

[6] Paccou J, Tsourdi E, Meier C, et al. Bariatric surgery and skeletal health: a narrative review and position statement for management by the European Calcified Tissue Society (ECTS). Bone 2022 154 116236. (10.1016/j.bone.2021.116236) 34688942

[7] Courtalin M, Verkindt H, Oukhouya Daoud N, et al. An evaluation of the implementation of the European Calcified Tissue Society recommendations on the prevention and treatment of osteoporosis secondary to bariatric surgery. Nutrients 2023 15 1007. (10.3390/nu15041007) 36839365 PMC9964124

[8] Shuhart C, Cheung A, Gill R, et al. Executive summary of the 2023 adult position development conference of the international society for clinical densitometry: DXA reporting, follow-up BMD testing and trabecular bone score application and reporting. J Clin Densitom 2024 27 101435. (10.1016/j.jocd.2023.101435) 38007332

[9] Lacombe J, Cairns BJ, Green J, et al. The effects of age, adiposity, and physical activity on the risk of seven site-specific fractures in postmenopausal women. J Bone Miner Res 2016 31 1559–1568. (10.1002/jbmr.2826) 26950269 PMC4973709

[10] Compston JE, Watts NB, Chapurlat R, et al. Obesity is not protective against fracture in postmenopausal women: glow. Am J Med 2011 124 1043–1050. (10.1016/j.amjmed.2011.06.013) 22017783 PMC4897773

[11] Yu EW, Lee MP, Landon JE, et al. Fracture risk after bariatric surgery: roux-en-y gastric bypass versus adjustable gastric banding. J Bone Miner Res 2017 32 1229–1236. (10.1002/jbmr.3101) 28251687 PMC5466471

[12] Mabeza RM, Mao Y, Maynard K, et al. Bariatric surgery outcomes in geriatric patients: a contemporary, nationwide analysis. Surg Obes Relat Dis 2022 18 1005–1011. (10.1016/j.soard.2022.04.014) 35589528

[13] Haentjens P, Magaziner J, Colón-Emeric CS, et al. Meta-analysis: excess mortality after hip fracture among older women and men. Ann Intern Med 2010 152 380–390. (10.7326/0003-4819-152-6-201003160-00008) 20231569 PMC3010729

[14] Roumpou A, Palermo A, Tournis S, et al. Bone in parathyroid diseases revisited: evidence from epidemiological, surgical and new drug outcomes. Endocr Rev 2025 46 576–620. (10.1210/endrev/bnaf010) 40177730 PMC12259238

[15] Liu Y, Côté MM, Cheney MC, et al. Zoledronic acid for prevention of bone loss in patients receiving bariatric surgery. Bone Rep 2021 14 100760. (10.1016/j.bonr.2021.100760) 33816718 PMC8005765

[16] Gam S, Lysdahlgaard S, Gram B, et al. Zoledronic acid increases spine bone mass and prevents hip bone loss after bariatric surgery: a randomized placebo-controlled study. Obesity 2025 33 659–670. (10.1002/oby.24214) 39978415 PMC11937863

[17] Flores LE, Mack L, Wichman C, et al. Protocol for a pilot randomised controlled trial of zoledronic acid to prevent bone loss following sleeve gastrectomy surgery. BMJ Open 2021 11 e057483. (10.1136/bmjopen-2021-057483) PMC8663101 34887285

